# Eltrombopag Modulates Reactive Oxygen Species and Decreases Acute Myeloid Leukemia Cell Survival

**DOI:** 10.1371/journal.pone.0126691

**Published:** 2015-04-27

**Authors:** Anna Kalota, Mary A. Selak, Laura A. Garcia-Cid, Martin Carroll

**Affiliations:** 1 Division of Hematology and Oncology, Perelman School of Medicine, University of Pennsylvania, Philadelphia, Pennsylvania, United States of America; 2 Department of Emergency Medicine, University of Pennsylvania, Philadelphia, Pennsylvania, United States of America; Università degli Studi di Firenze, ITALY

## Abstract

Previous studies have demonstrated that the small molecule thrombopoietin (TPO) mimetic, eltrombopag (E), induces apoptosis in acute myeloid leukemia (AML) cells. Here, we sought to define the mechanism of the anti-leukemic effect of eltrombopag. Our studies demonstrate that, at a concentration of 5 μM E in 2% serum, E induces apoptosis in leukemia cells by triggering PARP cleavage and activation of caspase cascades within 2–6 hours. The induction of apoptotic enzymes is critically dependent on drug concentration and the concentration of serum. This effect is not associated with an alteration in mitochondrial potential but is associated with a rapid decrease in a reactive oxygen species (ROS) in particular hydrogen peroxide (H_2_O_2_). Interestingly, E also decreases mitochondrial maximal and spare respiratory capacities suggesting an induced mitochondrial dysfunction that may not be readily apparent under basal conditions but becomes manifest only under stress. Co-treatment of MOLM14 AML cells with E plus Tempol or H_2_O_2_ provides a partial rescue of cell toxicity. Ferric ammonioum citrate (FAC) also antagonized the E induced toxicity, by inducing notable increase in ROS level. Overall, we propose that E dramatically decreases ROS levels leading to a disruption of AML intracellular metabolism and rapid cell death.

## Introduction

Eltrombopag (E) has been developed and tested as a small molecule thrombopoietin (TPO) mimetic and is FDA approved in the United States for the treatment of chronic immune (idiopathic) thromobocytopenia (ITP) and chronic hepatitis C associated thrombocytopenia [1–4]. This action is related to the ability of E to bind to and activate the c-Mpl protein, the endogenous receptor for TPO[5]. We and others subsequently showed that E and other related molecules are toxic to both leukemic and non-leukemic cell lines and to primary leukemic cells in vitro[5–8]. Surprisingly, this toxicity, unlike the platelet growth-stimulating effect of the drug, is independent of c-Mpl expression[9]. Thus, E has at least two discrete functions working through discrete mechanisms. The molecular events whereby E induces leukemic and cancer cell death are poorly defined.

Reactive oxygen species (ROS) encompasses a group of chemical entities that include hydrogen peroxide (H_2_O_2_), hydroxyl radical and superoxide anion. There are two major sources of superoxide anion in cells—the NADPH dependent oxidases (NOX) and the mitochondrial electron transport chain. Superoxide anion occurs as a byproduct of inefficient or disrupted electron transport during oxidative phosphorylation, and is rapidly converted to hydrogen peroxide through the action of superoxide dismutase (SOD). H_2_O_2_ in turn can be metabolized through several different pathways. The Fenton reaction uses Fe^+3^ as a catalyst to generate hydroxyl radical. In myeloid cells, myeloperoxidase uses H_2_O_2_ as a substrate to produce hypochlorous acid (HOCl) as part of the respiratory burst induced during phagocytosis. Additionally, several enzymes including glutathione peroxidase (GPx), catalases (CAT) and thiol peroxidases (TPx) can metabolize H_2_O_2_ into water. ROS are highly reactive species and their excess causes oxidative stress, leading to DNA and protein damage and eventually to a cell death [10–12] On the other hand, physiologic levels of ROS regulate a variety of cellular processes including cell cycle progression, cell motility, and growth factor signaling[13, 14]. Thus, it is important for the cell to control ROS homeostasis as the alternation of ROS levels either up or down leads to the activation of stress response. The amount of ROS necessary for normal cell function differs amongst cell types and depends on the cell metabolic state.

A hallmark of cancer cells compared to normal cells is a persistent pro-oxidative state that is a consequence of oncogenic transformation and/or alterations in metabolic activities leading to an intrinsic oxidative stress. Cancer cells have higher levels of reactive oxygen species (ROS) than normal cells, and ROS are, in turn, responsible for the maintenance of the cancer phenotype[15–18]. Addiction to high levels of ROS makes cancer cells more sensitive to disruption of homeostasis of those species. Our studies of E demonstrate that the drug dramatically decreases ROS level in leukemia cells, which results in tumor cell toxicity. Thus, we propose a novel mechanism of E’s antileukemic effect by alternation of ROS metabolism.

## Materials and Methods

### Reagents

Eltrombopag was provided by GlaxoSmithKline (Collegeville, PA, USA). Antimycin (AA), carbonyl cyanide 3-chlorophenylhydrazone(CCCP), L-buthionine-S,R-sulfoximine (BSO), hydrogen peroxide (H_2_O_2_), diphenylene iodonium (DPI), and iodoacetate (IAA) were purchased from Sigma-Aldrich (St. Louis, MO, USA). Other reagents were obtained as follows: N-acetyl-L-cysteine (EMD Millipore, Billerica, MA, USA). Tempol and NADPH (Tetrasodium Salt) (Santa Cruz Biotechnology, Santa Cruz, CA, USA).

### Cell culture

AML cell line MOLM14 was kindly provided by Dr. Donald Small at John Hopkins Medical Institute (Baltimore, MD, USA). AML cell lines: MV4-11, KG1a and HL-60 were obtained from American Type Culture Collection (ATCC) (Manassas, VA, USA). Primary samples were obtained from blood or bone marrow of healthy donors and patients with AML. All evaluated samples were previously collected, annotated and stored through an institutional review board (IRB) approved hematologic diseases tissue bank—Stem Cell and Xenograft Core (SCXC, ULR: http://www.med.upenn.edu/cores/stem_cell_and_xenograft.shtml) facility at the University of Pennsylvania. This Core is approved to collect and provide material from patients, after obtaining written informed consent, under University of Pennsylvania IRB protocol #70385. For this specific study all samples were anonymous and procured from SCXC core and therefore they fall into an exempt categories by federal regulation 45 CFR 46.101(b) and 21 CFR 56.104 and institutional IRB. No immortalized cell lines were generated from these samples. All cell lines used in this work have been previously established[19]. Both cell lines and primary cells were cultured in RPMI medium supplemented with 2% fetal bovine serum (FBS) or as indicated. Media and serum were purchased from Life Technologies (Grand Island, NY, USA). For proliferation assay, cell lines were plated at 5x10^4^ per 250 μL of culture medium and counted every day using trypan blue exclusion. Primary AML cells were plated at concentration 10x10^5^ cells per 250 μL of medium and counted every other day or as indicated.

### Western blot

Cells were plated at concentration 5x10^5^ per 1 mL of culture medium and incubated with/without drugs for indicated times. Then cells were washed once in phosphate buffer (PBS, Life Technologies, Grand Island, NY, USA), lysed and separated on SDS-PAGE 10% gel. Proteins were transferred to a PVDF membrane using semi-dry transfer apparatus (Bio-Rad, Hercules, CA, USA). The membrane was incubated overnight at 4°C with anti-Caspase 3, 9, 8 or PARP primary antibodies (Cell Signaling Technology, Danvers, MA, USA) and then with IRDye conjugated secondary antibodies. Signal was detected using Li-Cor imaging system (Lincoln, Nebraska USA) according to manufacturer’s directions.

### Quantitative real time PCR (Q-PCR) analysis

The Q-PCR analysis was performed as previously published [20] using following primers:

*DITT3* Forward: AGT CAT TGC CTT TCT CCT TCG GGA, Reverse: AAG CAG GGT CAA GAG TGG TGA AGA; *GADD45A* Forward: AAG GAT GGA TAA GGT GGG G, Reverse: CTG GAT CAG GGT GAA GTG G; *GADD45G* Forward: GCCGGCGTCTACGAGTCA Reverse: CCAGCACACAGAAGGTCACATT; *REDD1* Forward: TGTTTAGCTCCGCCAACTCT, Reverse: CACCCCAAAAGTTCAGTCGT; *ATF3* Forward: CCGAACTTGCATCACCA GTGC, Reverse: GAGCTGTGCAGTGCGCGCC; and *ACTIN* Forward: AAACTGGAACGGTGAAGGTG, Reverse: AGAGAAGTGGGGTGGCTTTT.

Quantitative analysis of PCR data employed the 2^-ΔΔCT^ method [21]

### Annexin V and BrdU staining

Cells were incubated with or without E for 4–12 h and then stained with ApoAlert Annexin V (Clontech, Mountain View, CA, USA) in accordance to the manufacturer’s protocol or with FITC BrdU Flow Kit (BD Biosciences, San Jose, CA, USA). For BrdU detection control cells and cells treated with E were pulsed-labeled with 10μM BrdU for 45 minutes then stained in accordance to the manufacturer’s protocol. After staining cells were analyzed by FACSCalibur flow cytometer (BD Biosciences, San Jose, California, USA). Collected data were analyzed using FlowJo software v.10.0.4.

### ROS detection and mitochondria membrane potential analysis

Cells were incubated with E or various mitochondrial electron transport chain and NADPH inhibitors for indicated time intervals. Cells were then washed once in PBS and loaded with carboxy-H_2_DCFDA, DHE or TMRE in accordance with the manufacturer protocol (Life Technologies, Grand Island, NY, USA) for measurements of intracellular levels of H_2_O_2_, O_2_^-^ and mitochondria membrane potential, respectively. After staining cells were analyzed by FACSCalibur flow cytometer (BD Biosciences, San Jose, California, USA). Collected data were analyzed using FlowJo software v.10.0.4. Extracellular levels of H_2_O_2_ were measured using AmplexRed (Life Technologies, Grand Island, NY, USA) assay in accordance with the manufacturer’s protocol.

### Oxygen consumption measurement

Oxygen consumption was measured using a Strathkelvin oxygen electrode in a magnetically stirred and thermostatically regulated chamber (37°C). Aliquots of cells were suspended in a total volume of 0.15 mL culture medium. The oxygen consumption rates are expressed as nanomoles O_2_ consumed/min/10^6^ cells.

### ATP production measurement

ATP production was measured using ATP bioluminescent somatic cell assay kit (Sigma-Aldrich, St. Louis, MO, USA) in accordance to manufacturer protocol using a Turner TD-20/20 luminometer (Sunnyvale, CA, USA). Briefly, MOLM14 cells were incubated with or without E for 20 hours. Both control and E treated cells were then split into 3 wells: 1) untreated, 2) IAA treated (100 μM for 2 hours), or 3) cells treated with antimycin AA (500 nM) + oligomycin O (1μg/mL) for 2 hours. Then cells were washed and put into a culture medium at concentration 1x10^6^/0.5 mL and ATP concentration was measured immediately.

## Results

### Eltrombopag Induces Rapid Cell Death in AML cell lines and primary cells

**Fig 1:**
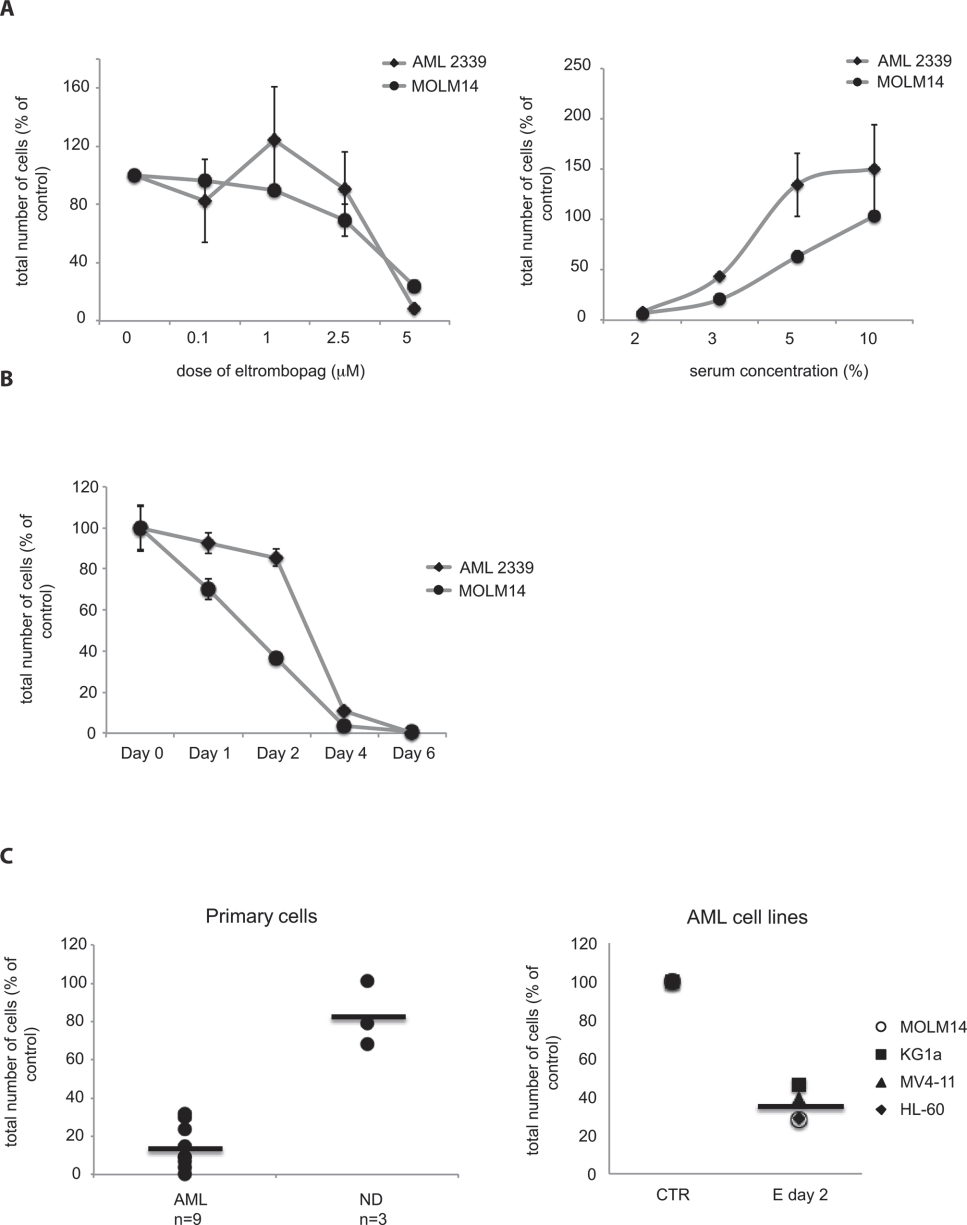
Cytotoxic effect of Eltrombopag on AML cells is dependent on dose and serum concentration. A) Dose response of primary AML cells and MOLM14 AML cell line (left panel) to E in culture medium containing 2% FBS and response to 5 μM E at the increasing concentration of serum in culture medium (2–10%) (right panel). Graphs represent cell counts at Day 4 for primary AML sample and Day 2 for MOLM 14 cells. Total number of viable cells in treated samples is presented as a % of total number of viable cells in control samples (untreated cells). Data are presented as an average of 3 independent experiments with a standard error of the mean (SEM). B) Time course of the response to 5 μM E in representative primary AML sample and MOLM14 cell line in 2% serum. C) Summary of the E’s effect on human primary AML cells n = 9 compared to peripheral blood mononuclear cells (PB-MNCs) from normal donors (ND) n = 3 (left panel) and AML cell lines n = 4 (right panel). Graphs demonstrate decrease in a total cell number after exposure to 5 μM E at 2% serum, presented as a percent of control at day 2 for cell lines and day 4 for primary cells.

To optimize culture conditions for studying the mechanisms of the antileukemic effect of E, we first evaluated the effects of dose of E and serum concentration on the cytotoxic effect of E. E elicited a concentration-dependent increase in cytotoxicity with cytotoxic concentration (CC_50_) observed between 2–3 μM in primary AML cells and 3–5 μM in AML cell lines, (Fig 1A left panel and Table 1) when cells were incubated in 2% serum. Interestingly, the E cytotoxicity was diminished with increasing concentration of serum in the culture medium (Fig 1A right panel), which may be partially due to protein binding of E. The cytotoxic effect of E at constant dose of 5 μM was detectable at serum concentrations below 5% for both cell lines and primary cells. The correlation between E dosage and cytotoxicity is particularly evident at day 2 of treatment for cell lines and day 4 for primary cells thus the CC_50_ was calculated at those days respectively (Fig 1B). These preliminary studies showed that the optimal condition to perform mechanistic studies was 5 μM E in culture medium containing 2% serum, a concentration sufficient to maintain cell viability and minimize drug binding to serum proteins. At these conditions we observed cytotoxic effect of E in 9 primary AML samples and 4 AML cell lines but not in peripheral blood mononuclear cells (PB-MNCs) from healthy donors (n = 3), which were significantly less sensitive to E treatment then AML cells (Fig 1C and Table 2).

**Table 1:** Effectiveness of E cytotoxicity presented as a half maximal growth inhibitory concentration (CC_50_).

**AML samples**	**CC50 of Eltrombopag in 2% serum at Day 4 (μM)**
1731	3.06
2293	2.15
1892	2.87
2339	2.68
3406	2.04
**AML cell lines**	**CC50 of Eltrombopag in 2% serum at Day 2 (μM)**
KG1a	5.4
MOLM14	4.9
MV4-11	3.6

The CC_50_ was calculated for primary AML samples (top) and AML cell lines (bottom) at days: 4 and 2 respectively, when cultured at 2% serum.

**Table 2:** Primary samples from patients with AML: patient characteristics.

Collection ID	Diagnosis	Subtype	Cytogenetics	SampleType	WBC	SEX	Age	Race
1731	AML	M5 (combining both M5a and M5b)	46,XY,t(3;9)(p14;p21)[20]	Pheresis	129	M	72.9	White
2293	AML	M4 [myelomonocytic]	46,XY[20]	Pheresis	195.5	M	51.6	White
1892	AML	NOS	46,XY[4]	Pheresis	90	M	N/A	white
2339	AML	NOS	N/A	Pheresis	349.0	M	47	White
3406	AML	AML-MLD with prior MPN	46,XY,del(20)(q11.2q13.1)[20]	Pheresis	170.4	M	66	White
3526	AML	AML with t(8;21)(q22;q22)	N/A	Pheresis	31.5	M	33	White
3516	AML	M1 [without maturation]	N/A	Pheresis	304.5	F	24	White
2522	AML	M4 [myelomonocytic]	complex *	Pheresis	113.0	M	52	White
2943	AML	M1 [without maturation]	46,XY[6]	Pheresis	193	M	62.7	White

complex*- 46,XY,add(6)(p21),del(8)(p21),add(12)(q24.1)[13]/46,XY,del(1)(q32),del(7)(q22q32),

der(6;12)(q10;p10),add(22)(q?11.2),+mar[3]/45,XY,t(1;2)(p?22;q11.2),-21[1]/46,XY[3]

N/A—not available

**Fig 2:**
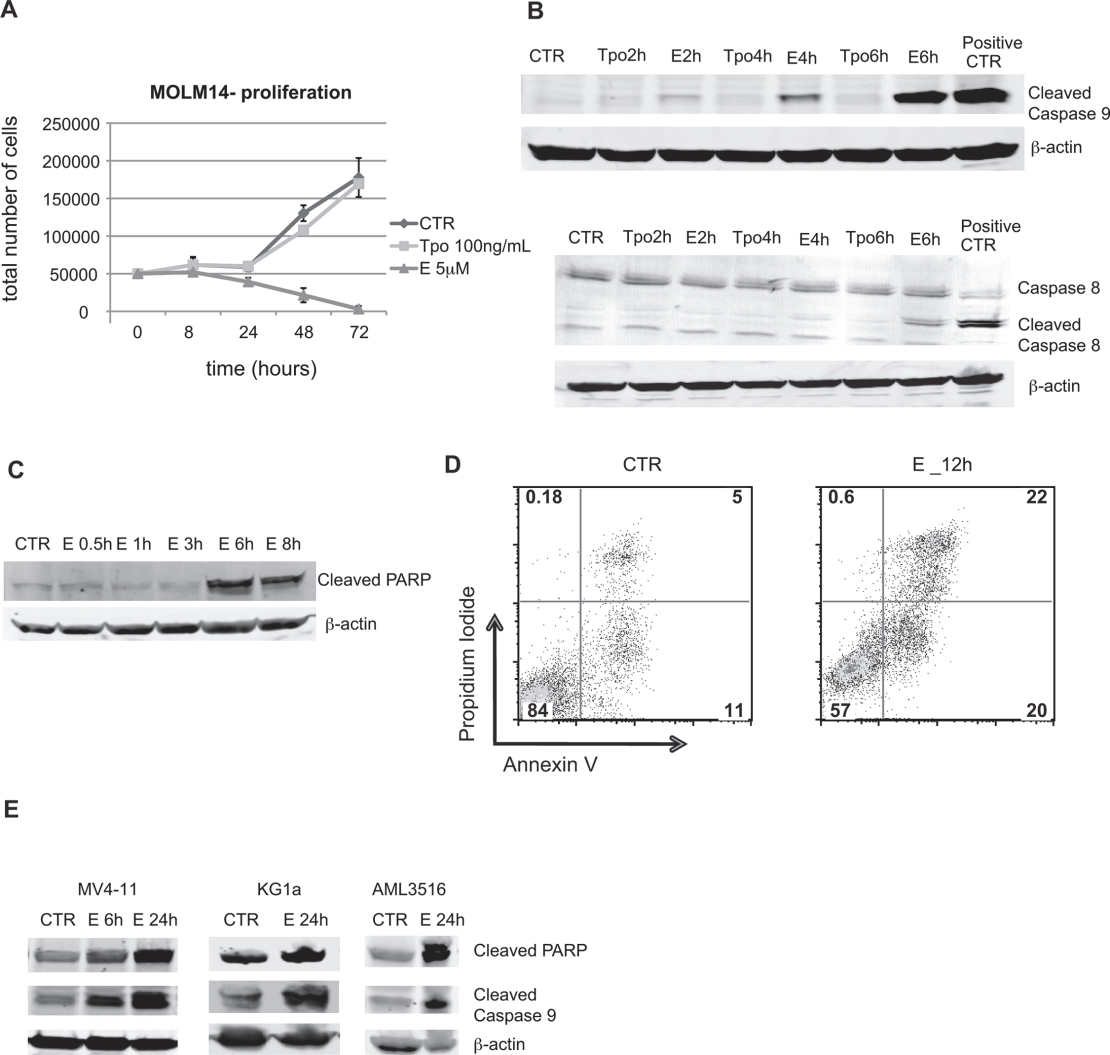
Activation of apoptotic pathway in AML cells by Eltrombopag. A) Graph represents time-course response of MOLM14 cells to E presented as an average of total number of viable cells from 3 independent experiments and SEM. B) Representative blots of Western blot analysis of activation of the apoptotic pathway at early time points (2–6 hours). Top panel—activation of Caspase 9, bottom panel—activation of Caspase 8. β- actin was used as a loading control. Positive CTR – cells incubated with etoposide 25 μM for 5 h. C) Western blot analysis of the activation of PARP protein. D) Representative flow cytometry plots of Annexin V and Propidium iodide staining of MOLM14 cells treated without or with E for 12 h. E) Western blot analysis of cleaved PARP and cleaved caspase 9 expression in additional AML cell lines MV4-11 (left panel) and KG1a (middle panel) and in primary AML cells (right panel). β-actin was used as a loading control. CTR—untreated control cells, E—cells treated with 5 μM E in the presence of 2% serum.

**Fig 3:**
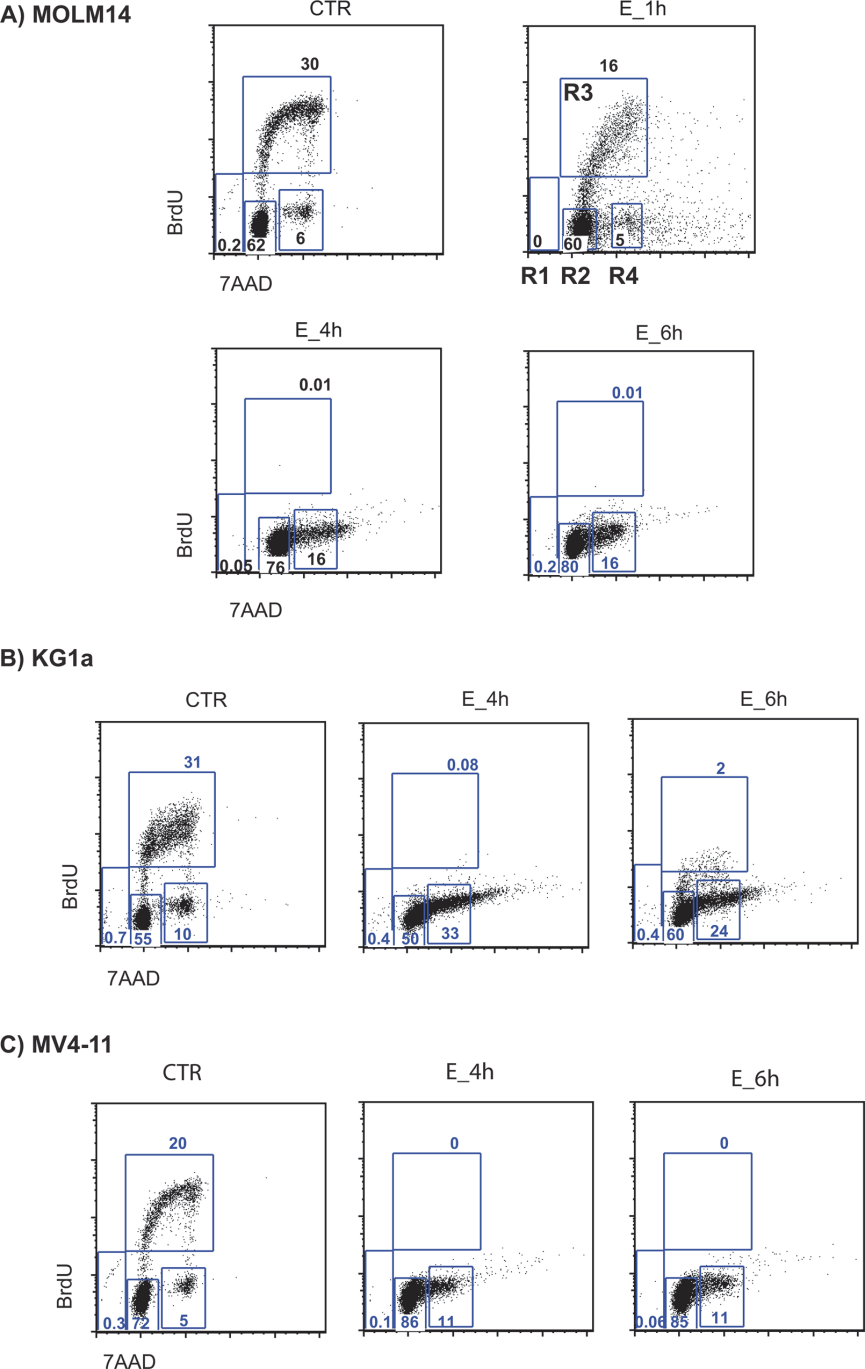
Eltrombopag completely inhibits S-phase DNA replication as measures by BrdU/7AAD staining in AML cells lines. Cell cycle analysis of A) MOLM14 B) KG1a, and C) MV4-11 AML cells treated without or with E assessed by BrdU/7AAD staining. Gates R1-R4 represent different cell cycle phases: R1—apoptotic cells, R2—G0/G1, R3—S phase and R4—G2+M. Control (CTR)—cells cultured in medium only, E- cells cultured in the presence of E (5 μM).

**Fig 4:**
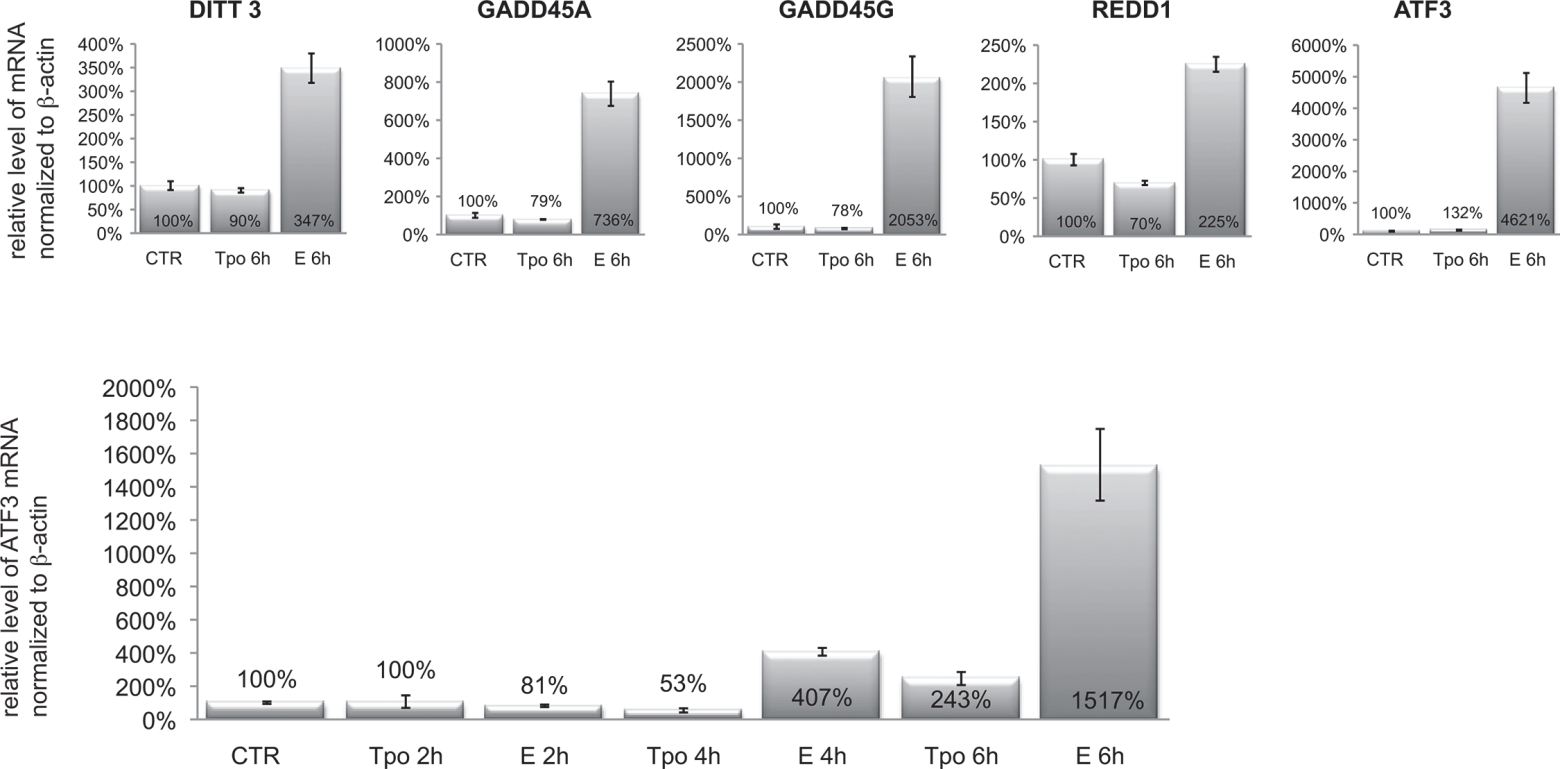
MOLM14 cells incubated with Eltrombopag for 6 hours show higher expression of stress response genes. Quantitative real time PCR (Q-PCR) measurement of mRNA level expression of five stress-response related genes in untreated cells (CTR), cells treated with rhTPO (100 ng/mL) and E (5 μM) for 6h (top panels) and earlier time point 2–6 h (bottom panel). Expression level of each gene is normalized to β-actin expression and presented as a percent of expression in untreated cells.

Subsequent kinetic studies with MOLM14 cells under these conditions showed that measureable cell death occurred within 24 hours of addition of 5 μM E (Fig 2A), suggesting a rapid induction of apoptotic pathways. As can be seen in Fig 2B (top), E-induced activation of caspase 9 by 2 hours after addition of E, and subsequent activation of caspase 8 (Fig 2B bottom) 3 and 7 (data not shown) and PARP by 6 hours (Fig 2C). E activated caspase 9 and PARP also in two other AML cell lines (MV4-11 and KG1a) and primary AML sample (Fig 2E). Activation of apoptotic pathway was confirmed by AnnexinV/PI staining. Differences between control and E treated cells were detectable after 12h (Fig 2D). Further, the cell cycle analysis by BrdU/7AAD staining showed decrease in S-phase of cell cycle as early as 1 hour after exposure to E and complete disappearance of cells in S-phase by 4 hours. At this time point most of the cells were accumulating at G1/G0 phase (Fig 3). No changes in BrdU/7AAD staining were observed between control and E treated cells in the presence of 10% serum (data not shown), which is consistent with the observation, that serum blocks cytotoxicity of E. Within 6 hours we also observed increased expression of stress response related genes including: *DITT3*, *GADD45A*, *GADD45G*, *REDD1* and *ATF3* (Fig 4). Because such rapid induction of cell death is seen in the cases of bioenergetic/redox/metabolic catastrophe such as mitochondrial depolarization or an effect on reactive oxygen species, we next examined the effect of E treatment on mitochondria and ROS.

### Eltrombopag Does Not Significantly Affect Mitochondria Function and ATP Production

**Fig 5:**
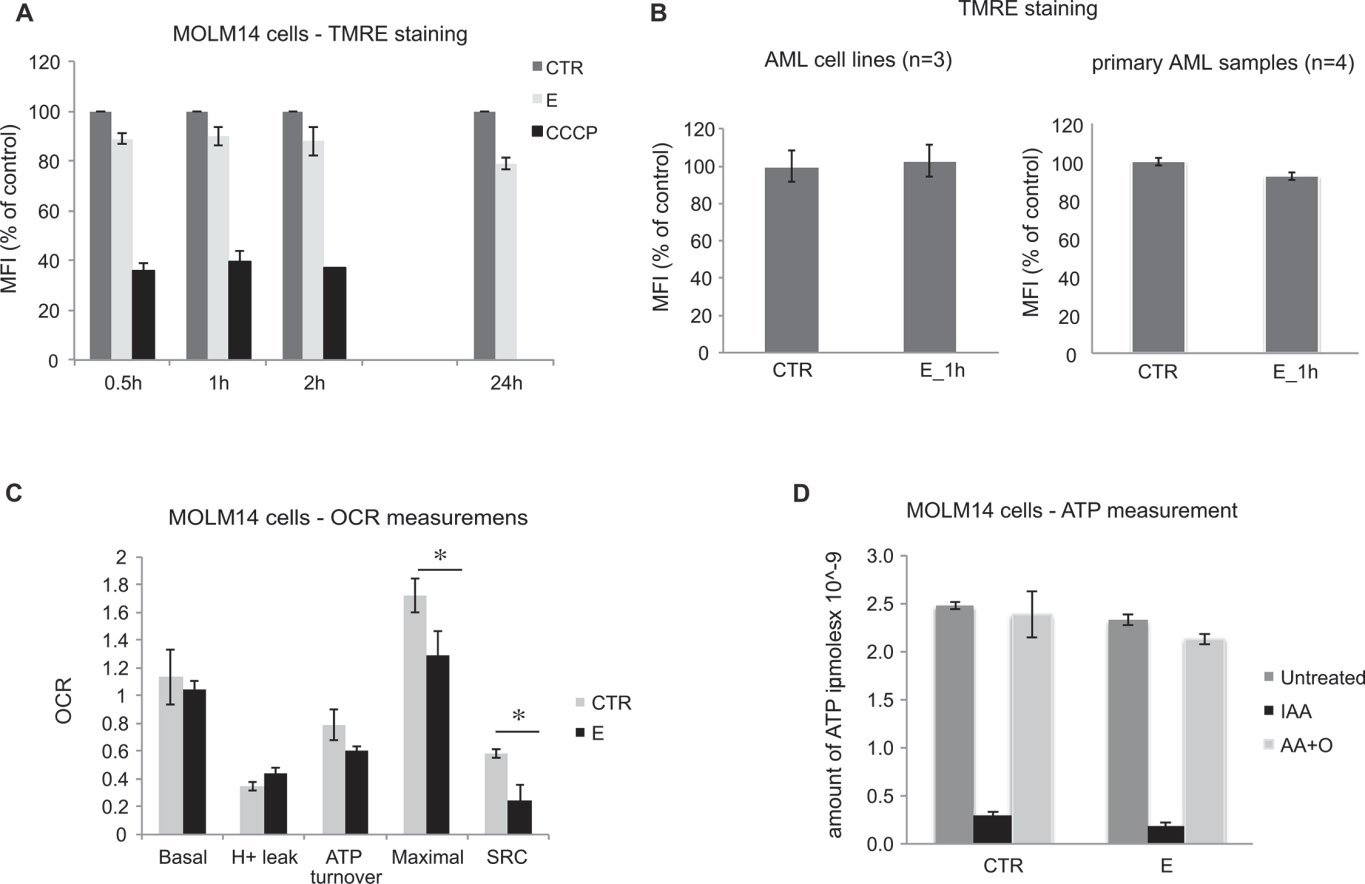
Eltrombopag Does Not Significantly Affect Mitochondria Function and ATP Production. A) Graph shows results of TMRE staining as a measurement of the mitochondria membrane potential in MOLM14 cells after exposure to E at various time points. CCCP, a known mitochondrial membrane potential disrupter was used as a control for the assay. Bars represent percent of control (untreated cells) mean fluorescence intensity (MFI) with SEM. B) Effect of E on mitochondrial membrane potential in AML cell lines (left panel) and primary AML cells (right panel) measured by a TMRE staining. C) Measurements of oxygen consumption rate in control and cells treated with E (5 μM) for 24 hours. The oxygen consumption rates are expressed as nanomoles O_2_ consumed/ min/ 10^6^ cells. The differences in maximal and spare respiratory capacity (SRC) between control and E treated cells are statistically significant with p value from 3 independent experiments equal 0.04 and 0.01 respectively. D) Graph represents an ATP production in MOLM14 cells cultured with or without (CTR) E for 20 hours, then exposed either to IAA, AA+O or remained untreated. Dark gray bars (untreated) represent total ATP, black bars (IAA) represent ATP produced by mitochondria and light gray bars (AA+O) represent ATP produced during glycolysis.

Given our apoptotic data, we initially sought to determine if E induces mitochondrial membrane depolarization. MOLM14 cells were incubated with E for 0.5 to 24 hours and assayed for mitochondrial membrane potential using TMRE, a cationic fluorescent dye that accumulates in energized mitochondria in response to an inside negative transmembrane potential. As seen in Fig 5A, E did not have a significant effect on TMRE accumulation in MOLM14 cells. No significant effect on mitochondrial membrane potential was further demonstrated in 3 additional AML cell lines (HL-60, MV4-11 and KG1a) and 4 primary AML samples (Fig 5B). These observations demonstrate that E does not induce depolarization of mitochondrial membrane potential but leaves open the possibility that E blocks mitochondrial electron transport.

Since the electron transport chain is responsible for more than 95% of all oxygen consumed by cells, oxygen consumption was used to examine the effect of E on mitochondrial function in intact cells. Sequential measurements were made of the basal respiratory rate, that following addition of the mitochondrial ATP synthase inhibitor oligomycin and maximal respiration stimulated by the uncoupler CCCP. Respiration supporting mitochondrial ATP synthesis was calculated by subtracting oligomycin-insensitive respiration from the basal respiration rate to yield oligomycin-sensitive oxygen consumption; oligomycin-insensitive respiration denotes proton leak across the inner mitochondrial membrane. Spare respiratory capacity (SRC) was calculated by subtraction of basal respiration from uncoupler-stimulated (maximal) respiration, a parameter indicating whether a bioenergetic reserve is available when demand for ATP is increased and thus shows whether or not a cell is likely to survive a bioenergetic/metabolic/oxidative stress. As can be seen in Fig 5C, E did not have a significant effect on basal, oligomycin-sensitive or oligomycin-insensitive oxygen consumption but significantly decreased both maximal oxygen consumption with uncoupler and the available SRC after 24 h exposure. The inhibitory effect of E on maximal respiration and SRC was also seen in primary AML cells (data not shown) demonstrating that this effect is not restricted to cultured cell lines. However, these effects of E on maximal oxygen consumption and SRC appear to be a late effect of the drug and not a primary cause of the rapid cell toxicity observed within 2–6 h.

AML cells utilize both electron transport and glycolysis to produce ATP. The balance between these two pathways varies amongst AML cell lines and reflects the various degrees to which the aerobic glycolysis occurs in AML cells[22]. We measured the effect of E on ATP production by mitochondria and through glycolysis in MOLM14 cells. E did not induce changes in either glycolytic or mitochondrial ATP production in these cells (Fig 5D).

### Eltrombopag Induces a Rapid Decrease in ROS levels

**Fig 6:**
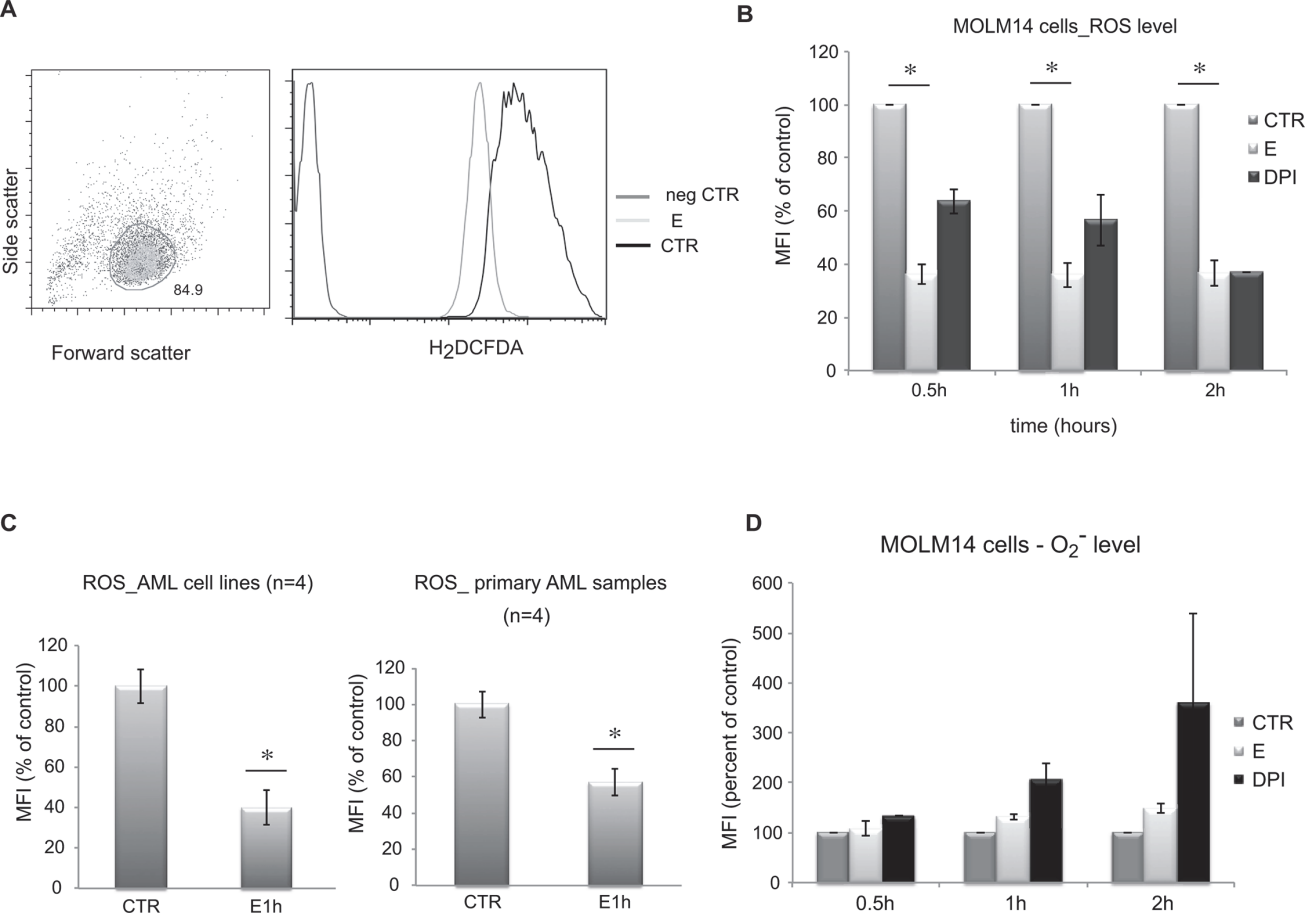
Eltrombopag markedly decreases levels of ROS in MOLM14 cells. Flow cytometry analysis of ROS in MOLM14 cells cultured with or without E. A) Representative flow charts show gating strategy (left panel) based on size (Forward Scatter) and granularity (Side Scatter) of the cells, and (central and right panel) Carboxy-H_2_DCFDA signal. B) Graphic presentation of ROS levels measured by flow cytometry in cells treated with E (5 μM) or DPI (25 μM), an inhibitor of NADPH oxidase known to decrease an intracellular level of ROS, or untreated (CTR) cells. Results are presented as an average of percent of control (untreated cells) mean fluorescence intensity with SEM. Data represent 4 independent experiments and the differences are statistically significant (*) p = 0.0000018. C) Relative level of ROS in AML cell lines: MOLM14, HL-60, MV4-11 and KG1a (left panel) and primary AML samples (right panel) after exposure to E. Differences between CTR and E treated cells are statistically significant (*) p = 0.0026 for cell lines and p = 0.001 for primary cells. D) Levels of O_2_^-^ in MOLM14 cells exposed to E measures at various time points using Dihydroethidium (DHE) stain. Control represents untreated cells, E—cells exposed to E (5 μM), DPI—cells exposed to DPI (25 μM). Data are presented as an average percent of control calculated based on mean fluorescence from 3 independent experiments and standard error of the mean.

We next asked if E affects total levels of reactive oxygen species. To perform this analysis, cells were incubated for 1–24 hours with E, washed and analyzed for total reactive oxygen species using carboxy-H_2_DCFDA dye, a common fluorescent probe for ROS. As shown in Fig 6A–6C E induces a rapid decrease in ROS in both AML cell lines and primary AML cells. This decrease, which is evident after just 0.5h-2h, persists for 24 hours (data not shown) in those cells that remain viable. We compared the effects of E to DPI compound. DPI is an inhibitor of NADPH oxidase, the other major source of ROS in cells. As shown in Fig 6B E decreased ROS more significantly than the NADPH inhibitor, suggesting either that E has more significant effects than DPI on ROS steady state. As DPI is a potent inhibitor of NADPH oxidase, the may suggest that E affects multiple pathways of ROS production.

**Fig 7:**
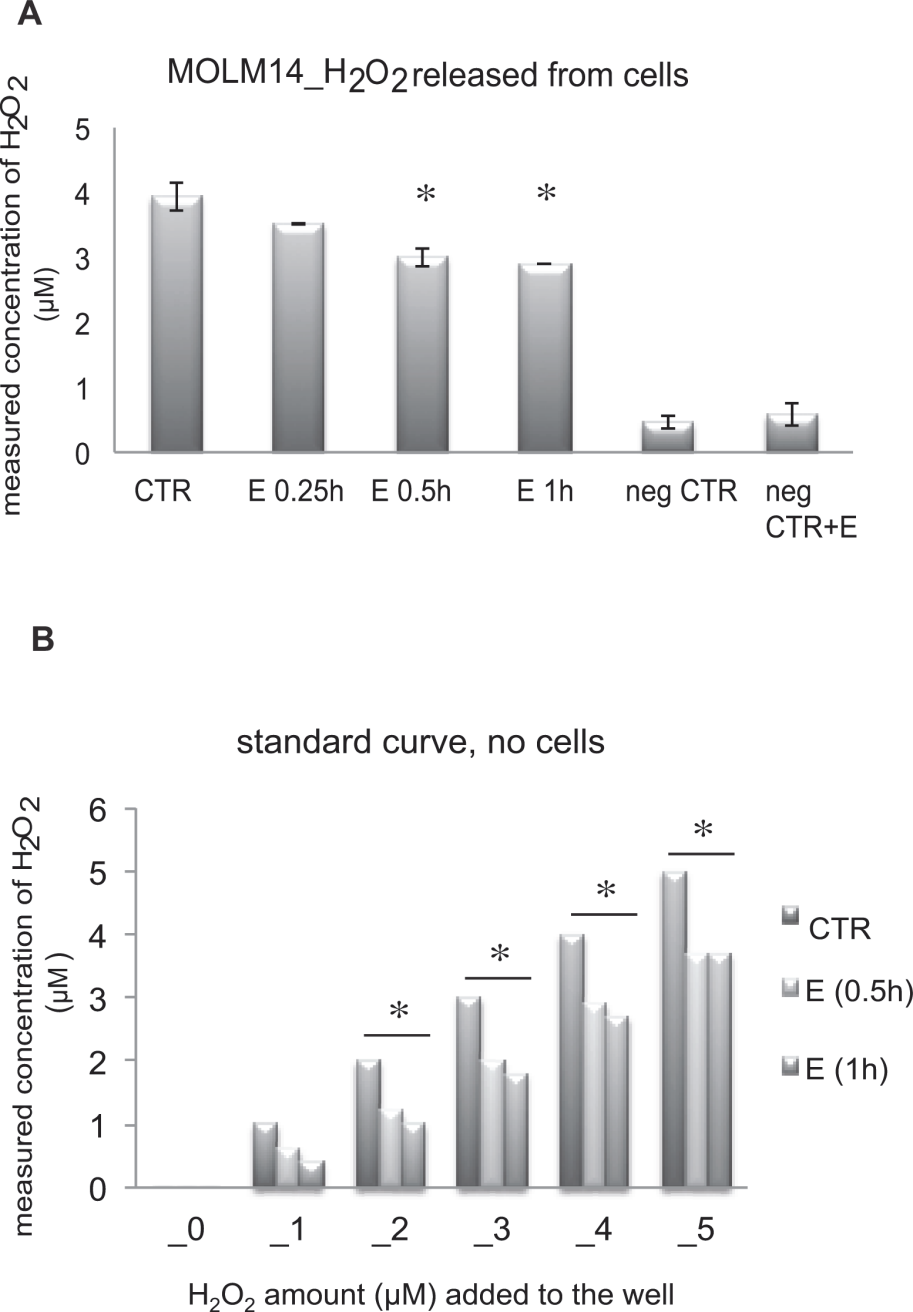
Amplex Red measurement of H_2_O_2_ released from MOLM14 cells correlates with the decrease in intracellular decrease of H_2_O_2_ measured using carboxy- H_2_DCFDA dye. A) H_2_O_2_ released from MOLM14 calculated based on the standard curve and presented in μM concentration. CTR—untreated cells, E—cells treated with 5 μM E, neg CTR—culture medium without cells, neg CTR+E—culture medium without cells + 5 μM E. B) Effect of E on H_2_O_2_ in the presence of hydrogen peroxidase and in the absence of cells. * p value < 0.05.

Carboxy-H_2_DCF reacts with multiple species of ROS including hydrogen peroxide (H_2_O_2_), superoxide anion and others. In order to determine the identity of the ROS species modulated by E, cells were treated as described for Carboxy-H_2_DCF but labeled with dihydroethidium (DHE), which fluoresces only after reaction with superoxide anion (O_2_^-^). No significant effect of E on O_2_^-^ was seen at 0.5–2 hours of incubation (Fig 6D). Because the decreases in Carboxy-H_2_DCF oxidation were evident within 30 min of incubation of MOLM14 cells with E, these results suggest that H_2_O_2_ might be the critical ROS species affected by E. We measured then the effect of E on H_2_O_2_ using Amplex red. Amplex Red is a fluorogenic substrate for horseradish peroxidase that in the presence of H_2_O_2_ is oxidized to produce the highly fluorescent compound resorufin and is thus specific for H_2_O_2_. Amplex Red is impermeable to cells and thus can be used to measure H_2_O_2_ released from the cells into the extracellular medium or present in solution. Consistent, with the carboxy-H_2_DCFDA results, Amplex Red measurements showed decreases in H_2_O_2_ released from MOLM14 cells exposed to E as compared to untreated cells (Fig 7A). Moreover, in vitro Amplex Red experiments showed that E directly decreases H_2_O_2_ levels in solution in the presence of hydrogen peroxidase (Fig 7B). Taken together, these results strongly suggest that H_2_O_2_ is likely the direct target of E in MOLM14 cells.

### Extent of eltrombopag cytotoxic effect correlates with level of ROS

**Fig 8:**
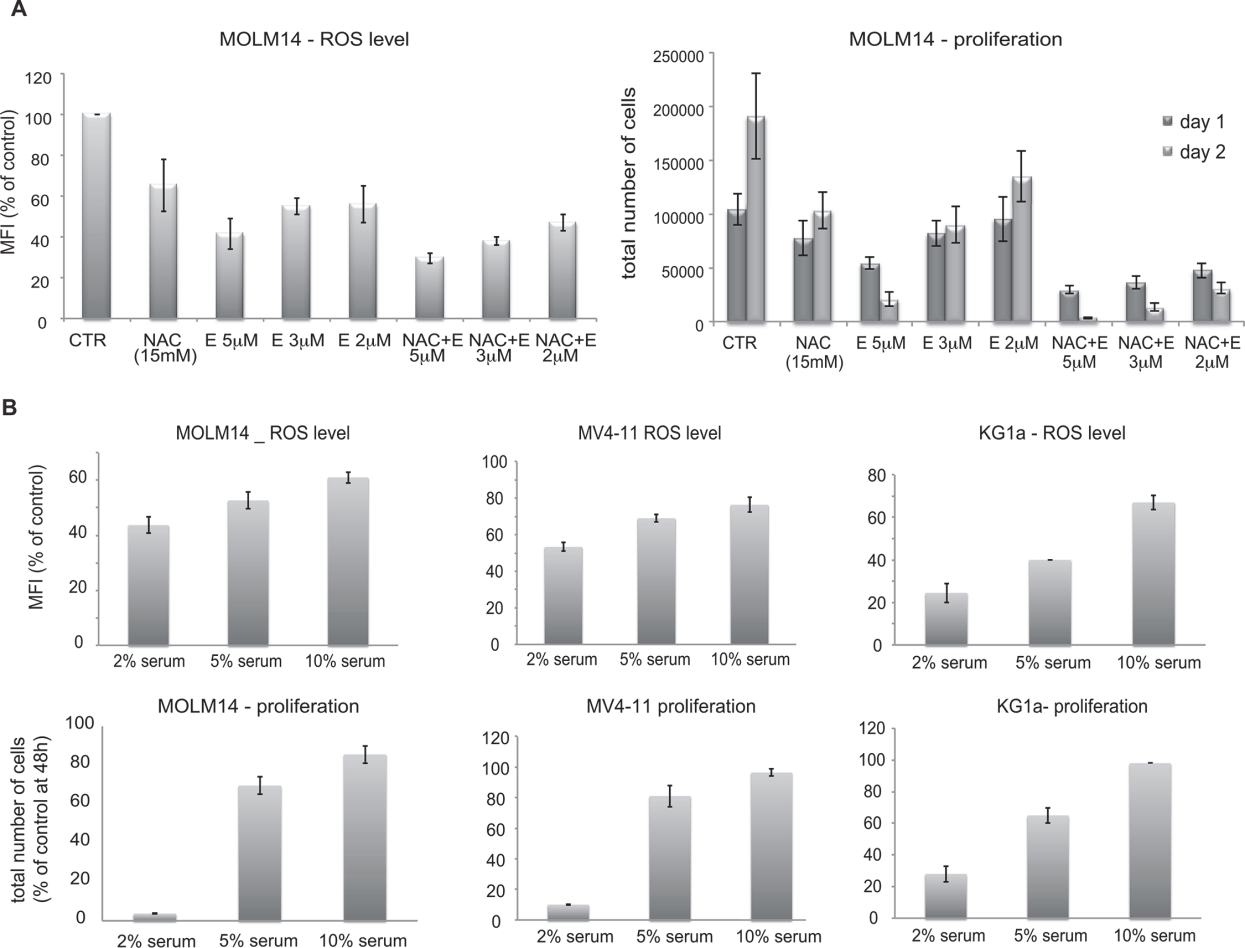
Correlation between eltrombopag’s cytotoxic effect and ROS level at various drug doses and serum concentrations. A) Additive effect of E combined with antioxidant (NAC) on ROS decrease and survival of MOLM14 cells. Left panel—levels of H_2_O_2_ measured using carboxy- H_2_DCFDA in MOLM14 cells exposed to E and NAC for 1 hour. Right panel—Proliferation of MOLM14 cells exposed to E (2–5 μM) alone NAC (15 mM) alone or combination. Results are presented as an average of total number of cells counted at days: 1 and 2 and SEM from 2 independent experiments. B) Increasing serum concentration decreases effect of E on ROS level (top panels) and cell survival (bottom panels) in AML cell lines. Results are presented as an average of percent of control of total number of cells counted at day 2 and SEM from 2 independent experiments.

In order to determine if altering ROS modulates the effect of E on AML cells, we treat MOLM14 cells with *N*-acetylcysteine (NAC), a known antioxidant in the presence and absence of E for 24 hours. As expected (Fig 8A left panel), both treatment with E and NAC decreased ROS in cells. E was significantly more potent than NAC and decreased ROS in a concentration-dependent manner between 2–5 μM. Addition of NAC (15 mM) further decreased ROS levels below the level elicited by 2 or 3 μM E alone. It is noteworthy that NAC also increased the cytotoxicity of low concentrations of E (Fig 8A right panel), consistent with the hypothesis that low ROS levels induce cell death in MOLM14 cells. We next investigated the effect of E on ROS in the presence of increasing serum concentration. Similar to the cytotoxic effect we found ROS level to also be less affected by E at higher serum concentration (Fig 8B). Together these data demonstrate clear correlation between cytotoxicity of E and intracellular level of ROS.

### Inducing ROS production partially rescues MOLM14 cells from E toxic effect

**Fig 9:**
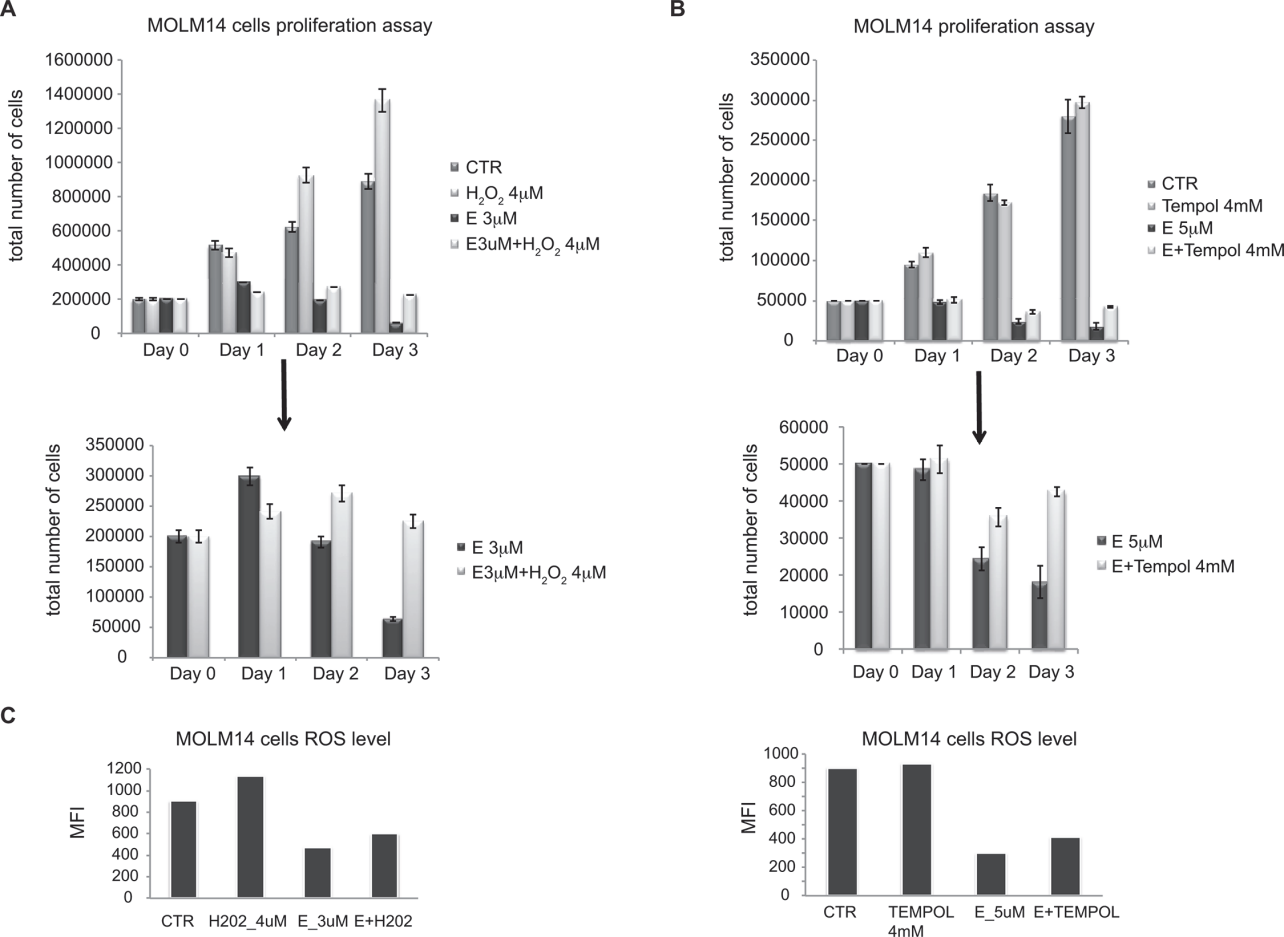
Effect of E is partially diminished by addition of H_2_O_2_ or SOD mimetic to augment ROS level. A) Proliferation of MOLM14 cells in the presence of E with and with or without addition of 4 μM H_2_O_2_. Top panel includes untreated cells (CTR) and cells treated with 4 μM H_2_O_2_ only, bottom panel is a magnification of bars representing cells treated with E alone and E+ H_2_O_2_. B) Proliferation of MOLM14 cells in the presence of E with or without SOD mimetic tempol (4 mM). Top panel includes untreated cells (CTR) and cells treated with 4 μM H_2_O_2_ only, bottom panel is a magnification of bars representing cells treated with E alone and E+Tempol. C) ROS levels in MOLM 14 cells cultured in the presence of E alone, H_2_O_2_ alone and E+ H_2_O_2_ (left panel) and E alone, tempol alone and E+ tempol (right panel) for 1h. ROS level is presented as a mean fluorescence intensity (MFI).

**Fig 10:**
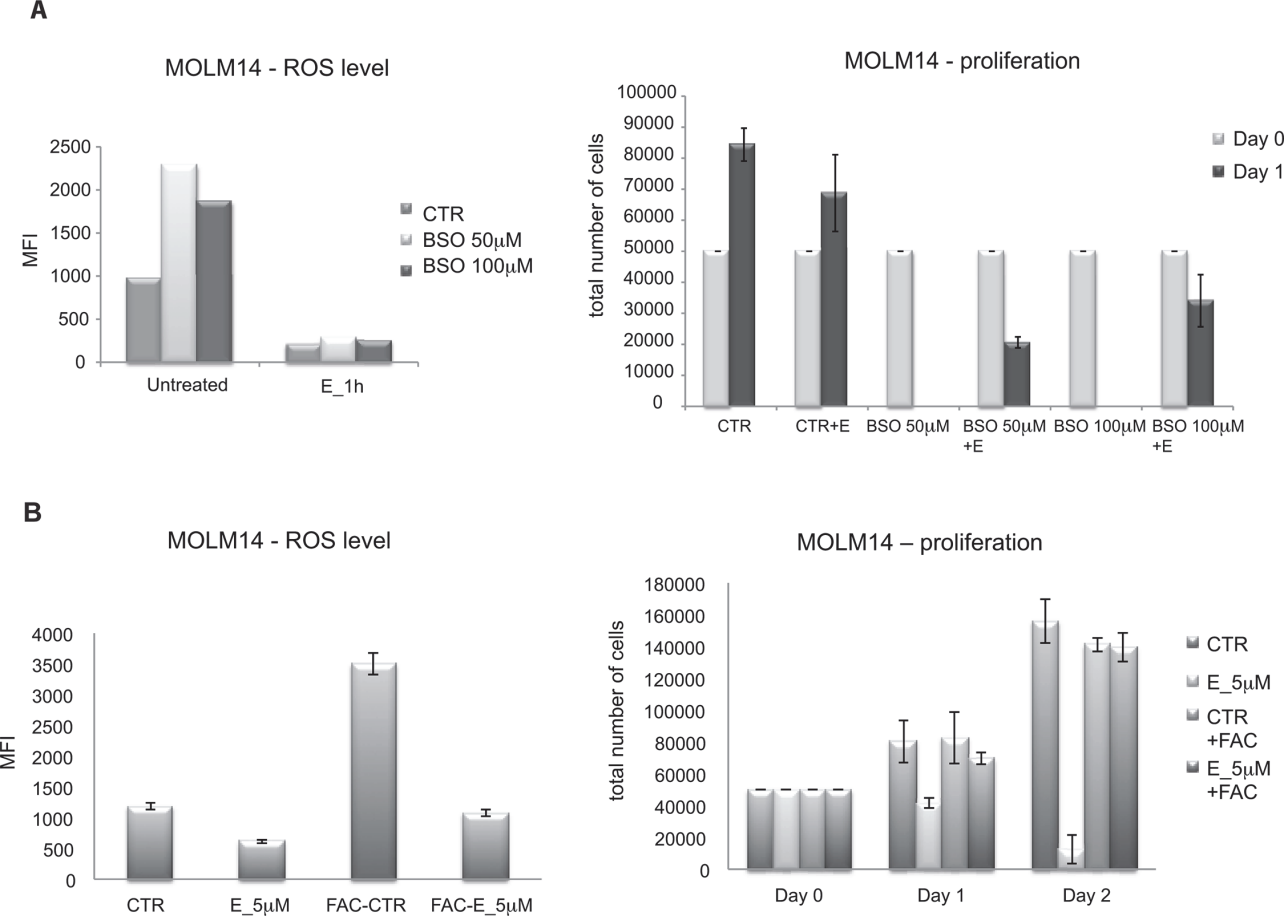
Modification of intracellular ROS level abrogates eltrombopag’s effect on AML cells. A) E blocks completely increase of ROS caused by BSO and rescues cells from BSO induced death. Left panel—ROS level in MOLM14 cells untreated (CTR) or pre-treated with BSO for 72 hours and then exposed to E or left untreated. Right panel—proliferation assay—results are presented as an average and SEM of a total number of cells from 3 independent experiments. At day 0 cells cultured in the presence of BSO (50 or 100 μM) for 72hours or in plain medium (CTR) were plated at concentration 5x10^4 per 250 μL of culture medium and exposed to E or left untreated. B) Pre-loading with ferric ammonium citrate (FAC) rescues MOLM14 cells from E cytotoxic effect by inducing ROS level. Levels of H_2_O_2_ measured using carboxy- H_2_DCFDA in MOLM14 cells treated as described above (left panel). Proliferation of MOLM14 cells un-manipulated or pre-loaded with 500 μg/mL of FAC for 24 h and then exposed to E or left untreated (CTR) (right panel).

Although, modifying H_2_O_2_ levels directly is challenging because of the poor chemical stability of the compound, we did attempt to rescue E-treated MOLM14 cells with H_2_O_2_. As shown in Fig 9A, cells treated with E showed cell death that increases over time. Exogenous addition of H_2_O_2_ to the media, particularly at 4 μM, partially rescued this effect. A further experiment was performed to test this hypothesis. Tempol is a SOD mimetic, which is anticipated to increase ROS. We tested the effect of Tempol on E treated cells for cell survival after E treatment. Cells treated with Tempol plus E survived better than cells treated with E alone (Fig 9B). In both experiments the rescue effect was partial, which correlated with modest increase in ROS level (Fig 9C). This is consistent with our hypothesis that the rapid decrease in ROS levels in E—treated cells leads to rapid apoptosis. In a complementary experiment, we blocked GSH synthesis by treating cells with BSO-(L-buthionine-S,R-sulfoximine), an inhibitor of gamma-glutamyl-cysteine synthetase. BSO caused an increase in ROS levels as expected (Fig 10A left panel). Pre-treatment of cells with E, completely blocked this effect. As noted, BSO is itself toxic and 50 μM BSO completely eliminated viable cells after 24 hours of treatment. This result is at first paradoxical but is consistent with the hypothesis that AML cells exist in an unstable metabolic state making them susceptible to either acute decreases or increases in ROS levels. Consistent with a primary effect on ROS, E when combined with BSO actually partially rescues the cells from BSO induced cell death (Fig 10A right panel). This demonstrates that the primary effect of E is on ROS and not toxicity through an unrelated cell mechanism.

### Effect of Fe^+3^ on eltrombopag effects is dependent on ROS modulation

The interaction between iron metabolism and ROS is complex and complementary. Recently, Roth et.al performed an experiment to determine the role of iron in E effects [7]. These investigators incubated cells in E without or with ferric ammonium citrate (FAC), which provides a source of Fe^+3^. Interestingly, this experiment rescued cells from the toxic effects of E. We have confirmed that FAC rescues MOLM14 cells from toxic effects of 5 μM E in 2% serum. We pursued this further by examining the effect of FAC on ROS. Cells were incubated in FAC alone, E alone or the combination. As shown (Fig 10B), E decreased ROS as previously observed but FAC greatly increased ROS in cells. The combination led to a cumulative no effect on ROS. Overall, these data indicate to us that FAC rescues cells from the toxic effects of E by inducing ROS.

## Discussion

We have previously demonstrated the cytotoxic effects of E on primary human AML cells and various cancer cell lines[9]. Here we show that these cytotoxic effects are tightly associated with serum concentrations. E is toxic at serum concentrations between 2–10% but the concentration of E required to induce rapid apoptosis of cells increases with increasing serum concentration in this range. We hypothesize that this effect is due to protein binding as incubation of cells in serum free media with 5% bovine serum albumin (BSA) (data not shown) added also decreases the apoptotic effects of E. Given this observation and the observation that it is difficult to adapt MOLM14 cells to serum free conditions, we have chosen to perform experiments at 2% fetal bovine serum with 5 μM E as our standard conditions. We believe that use of this low serum condition allows the “cleanest” conditions for definition of the mechanism of action. Under these conditions, E decreases hydrogen peroxide levels and the induction of cell death correlates with this decrease. We propose that a decrease in H_2_O_2_ level is a necessary step in E—induced cell toxicity.

Recent phase I clinical trials that evaluated the safety and efficacy of E monotherapy in patients with AML showed that E is well tolerated at doses up to 4 times higher then the dose currently approved by the FDA for ITP treatment. The plasma concentration of E following FDA approved dose (up to 75mg daily) has been reported to be ~ 10 μg/mL[23]. The 4 fold higher doses evaluated in clinical trial conducted at UPENN yielded plasma concentration exceeding 100 μg/mL[24], which is equivalent to 18 μM a dose much higher then the concentration used in our experiments. Such high dose was toxic to AML cell lines in the presence of 10% serum (data not shown). Moreover, one subject who received the highest dose of E achieved complete response and remains on the drug[25]. These data suggest that conditions used in our experiments are clinically achievable. Further studies will be required to determine the in vivo effect of E on H_2_O_2_ levels of AML cells.

Understanding the mechanisms of E’s anti-leukemic effect has been a challenging task considering the chemical structure of the molecule, which makes it highly prone to oxidative metabolism[26]. Our studies demonstrate that E induces a cell cycle arrest and a rapid apoptotic response in cells within 2–4 hours of incubation. E does not induce significant changes in mitochondrial function on a time scale adequate to explain this rapid toxicity, but in contrast induces a rapid decrease in ROS levels that are likely to reflect a decrease in H_2_O_2_. Treatment of cells with H_2_O_2_ itself or with SOD mimetic—Tempol to increase ROS levels overcomes the toxicity of E on MOLM14 cells. On the other hand, E augments the toxic effect of an antioxidant NAC on AML cell lines. Taken together our data strongly suggest that E rapidly decreases the H_2_O_2_ level, which leads to inhibition of cell cycle and eventually to an AML cell death.

Of note, however, Roth and colleagues recently reported that E inhibits cells proliferation of leukemia cells by reduction of intracellular level of iron [7]. In their conditions with 5 μg/mL (~10 μM) of E in 10% serum, E inhibited proliferation and induced differentiation of leukemic cells within 72 hours. In contrast with our observations they noticed increase in ROS after exposing cells to E for 1h as measured using H_2_DCFDA dye. Given this discrepancy, we have spent considerable effort trying to clarify these differences. In our experiments we have confirmed E-induced decreases of ROS using two different fluorescent indicators: carboxy-H_2_DCFDA and Amplex red. We have demonstrated clear correlation between decrease in ROS and cytotoxic effect, which was dependent on dose of E and the concentration of serum in culture medium. The fact that E decreased H_2_O_2_ level in a cell free system in the presence of hydrogen peroxidase as measured by Amplex red and ability of E to diminish inducing effect of BSO on ROS production demonstrate that observed decrease in ROS is a real effect.

To determine whether the primary effect of E on leukemia cells is due to a decrease of iron, as suggested by Roth et al.[7] or by decrease in ROS remains to be determined. More precise biochemical analysis is required to answer such questions. Considering the fact that iron acts as a catalyzer in Fenton reaction to decompose H_2_O_2_, it is likely that these two processes are tightly linked.

In conclusion we propose a novel mechanism of E’s toxic effect on leukemia cells. Our studies demonstrate that E induces AML cell death by a rapid decrease in H_2_O_2_ level and combination of E with reagents to further stress the metabolic stability of AML cells by, for example, decreasing cellular glutathione levels or chelating iron, should lead to augmentation of the effects of E in inducing AML cell death. These studies thus suggest ways to further develop E as an anti-leukemic drug by combining the compound with other agents.
